# Investigation of multidrug-resistant plasmids from carbapenemase-producing *Klebsiella pneumoniae* clinical isolates from Pakistan

**DOI:** 10.3389/fmicb.2023.1192097

**Published:** 2023-06-29

**Authors:** Christine Lascols, Blake Cherney, Andrew B. Conley, Lavanya Rishishwar, Matthew A. Crawford, Stephen A. Morse, Debra J. Fisher, Kevin Anderson, David R. Hodge, Segaran P. Pillai, Molly A. Hughes, Erum Khan, David Sue

**Affiliations:** ^1^National Center for Emerging and Zoonotic Infectious DiseasesCenters for Disease Control and Prevention, Atlanta, GA, United States; ^2^IHRC, Inc., Atlanta, GA, United States; ^3^Division of Infectious Diseases and International Health, Department of Medicine, University of Virginia, Charlottesville, VA, United States; ^4^Science and Technology Directorate, U.S. Department of Homeland Security, Washington, DC, United States; ^5^Office of the Commissioner, U.S. Food and Drug Administration, Silver Spring, MD, United States; ^6^Department of Pathology and Laboratory Medicine, Aga Khan University, Karachi, Pakistan

**Keywords:** *Klebsiella pneumoniae*, AMR determinants, MDR plasmids, nanopore hybrid assemblies, AMR data analysis

## Abstract

**Objectives:**

The study aim was to investigate multidrug-resistant (MDR) plasmids from a collection of 10 carbapenemase-producing *Klebsiella pneumoniae* clinical isolates identified within the same healthcare institution in Pakistan. Full characterization of the MDR plasmids including structure, typing characteristics, and AMR content as well as determination of their plasmid-based antimicrobial susceptibility profiles were carried out.

**Methods:**

Plasmids were isolated from 10 clinical isolates of *Klebsiella pneumoniae*, and from a corresponding set of *Escherichia coli* transconjugants, then sequenced using Nanopore/Illumina technology to generate plasmid hybrid assemblies. Full characterization of MDR plasmids, including determination of next generation sequencing (NGS)-based AMR profiles, plasmid incompatibility groups, and types, was carried out. The structure of MDR plasmids was analyzed using the Galileo AMR platform. For *E. coli* transconjugants, the NGS-based AMR profiles were compared to NGS-predicted AMR phenotypes and conventional broth microdilution (BMD) antimicrobial susceptibility testing (AST) results.

**Results:**

All carbapenemase-producing *K. pneumoniae* isolates (carrying either *bla*_NDM-1_, or/and *bla*_OXA-48_) carried multiple AMR plasmids encoding 34 antimicrobial resistance genes (ARGs) conferring resistance to antimicrobials from 6 different classes. The plasmid incompatibility groups and types identified were: IncC (types 1 and 3), IncFIA (type 26) IncFIB, IncFII (types K1, K2, K7, and K9), IncHI1B, and IncL. None of the *bla*_NDM-1_ and *bla*_ESBL_-plasmids identified in this study were previously described. Most *bla*_NDM-1-_plasmids shared identical AMR regions suggesting potential genetic material/plasmid exchange between *K. pneumoniae* isolates of this collection. The majority of NGS-based AMR profiles from the *E. coli* transconjugants correlated well with both NGS-based predicted and conventional AST results.

**Conclusion:**

This study highlights the complexity and diversity of the plasmid-based genetic background of carbapenemase-producing clinical isolates from Pakistan. This study emphasizes the need for characterization of MDR plasmids to determine their complete molecular background and monitor AMR through plasmid transmission between multi-resistant bacterial pathogens.

## Introduction

Bacterial infections caused by multidrug resistant (MDR) pathogens represent a major public health threat that jeopardizes antimicrobial therapy and its fundamental role in modern medicine (World Health Organization, 2014; CDC Report, 2019). The transfer of antimicrobial resistance (AMR) genes between bacteria of the same or different species often occurs via mobile genetic elements (MGE), such as plasmids, during horizontal gene transfer (HGT). Strategies to combat AMR include rapidly identifying MDR pathogens, elucidating disease transmission pathways for community-acquired and nosocomial infections and implementing effective prevention and infection control measures.

The continued emergence and global spread of ESBLs and carbapenemases among MDR pathogens responsible for life-threatening infections are alarming (Castanheira et al., 2019). ESBL- and carbapenemase-producing bacteria often carry additional AMR determinants that confer resistance to other classes of antimicrobials (Mączyńska et al., 2023). The 2018 isolation of a *Klebsiella (K.) pneumoniae* strain in the United States that was resistant to all currently available antibiotics, highlights the challenges of treating infections caused by “pan-resistant pathogens” (de Man et al., 2018).

High fidelity NGS data are critical for accurate plasmid characterization and the identification of ARG variants necessary to predict AMR profiles with high concordance to phenotypic AST results in bacteria (Neuert et al., 2018; Yee and Simner, 2022). The portable MinION (Oxford Nanopore Technologies; ONT) long-read sequencer addresses the limitations observed with many NGS technologies for plasmid sequencing (e.g.; abundance of repetitive elements), and offers a potential solution in real-time and at a much lower cost (e.g., ~$2 K for a start-up kit) for reconstructing MGEs (e.g.; plasmids) and facilitating the identification of ARGs while also helping to track plasmid dissemination and the spread of AMR (Brown et al., 2023).

Since most ARGs are located on plasmids and transmitted to other microorganisms through HGT, long-read plasmid sequencing is essential for characterizing mechanisms of resistance, and understanding the basis of plasmid dissemination, and for tracking MDR pathogens during epidemiological surveillance studies in hospital and community settings (Hendriksen et al., 2019; Hadjadj et al., 2022; Valcek et al., 2022; Yang et al., 2022). Plasmid sequence data from the original strain is used as starting material, and the plasmid of interest can then be transferred to a well-characterized bacterial strain [e.g., *Escherichia (E.) coli* J53] for further comprehensive analysis. Although this approach requires additional laboratory work, there are potential benefits. Following transformation of a host strain, the plasmid-mediated contributions to AMR transmission are clarified since the chromosome of the original strain is absent. Also, transferring a single plasmid allows the study of the direct phenotypic effects from the ARGs located on that specific plasmid.

Since the first identification of NDM-positive clinical *Enterobacteriaceae* isolates in a patient from Sweden who visited India in 2009, NDM has quickly spread worldwide due to its location on MGEs, and continuous surveillance and characterization of MGEs in regions around India where NDM originated such as Pakistan is essential for implementing control measures in hospitals and community.

The collection of 10 carbapenemase-producing (i.e., NDM- and/or OXA-48) MDR *K. pneumoniae* clinical isolates from Pakistan described in our previously published study (Lomonaco et al., 2018) was randomly selected with at least resistance to one carbapenem. Multiple STs were identified: ST11 (*n* = 3), ST14 (*n* = 3), ST15 (*n* = 1), ST101 (*n* = 2), and ST307 (*n* = 1), and identical AMR content was observed for some isolates with the same ST. The aim of the current research was to further investigate and fully characterize MDR plasmids to identify potential transmission of the same plasmids or plasmids rearrangements, between *K. pneumoniae* clinical isolates in this same hospital in Pakistan. Unlike our first study using Illumina sequencing which is not suitable for plasmids, ONT (long-read sequencing) was combined with Illumina (short-read sequencing) to generate long hybrid assemblies and successfully resolve MDR plasmids. We characterized the *bla*_NDM-1_ and *bla*_OXA-48-_plasmids and identified the genetic origin of AMR for 10 MDR *K. pneumoniae* isolates using free publicly available software tools. Using these hybrid assemblies, we: (1) determined plasmid incompatibility groups and types, (2) determined NGS-based resistance profiles and compared with NGS- and AST-phenotypes from *E. coli* transconjugants (3) fully characterized the genetic structure of *bla*_NDM-1_ and *bla*_OXA-48_-plasmids. This study aimed to determine genetic relatedness and transferability of AMR plasmids within this collection of clinical isolates to help monitor AMR transmission in hospitals, which constitute an important healthcare problem.

## Materials and methods

### Bacterial isolates

Ten MDR *K. pneumoniae* isolates, isolated from patients in 2013 and identified as carbapenemase-positive harboring either *bla*_NDM-1_ (*n* = 5), *bla*_OXA-48_ (*n* = 3), or both (*n* = 2), were obtained from the Clinical Microbiology Laboratory at the Department of Pathology and Laboratory Medicine, Aga Khan University Hospital, Karachi, Pakistan. The 10 *K. pneumoniae* isolates were recently described in our previous work: BL849 (CFSAN044563), BU19801 (CFSAN044564), MS84 (CFSAN044565), BL12125 (CFSAN044566), BL12456 (CFSAN044568), BA3783 (CFSAN44569), BL13802 (CFSAN044570), BA2664 (CFSAN044571), BL8800 (CFSAN044572), and BA2880 (CFSAN044573). BL12125 (CFSAN044566), BL12456 (CFSAN044568) including two pairs of isolates closely related to each other and sharing the same AMR profile: BL12125 (CFSAN044566), and BL12456 (CFSAN044568) belonging to ST14, and BL8800 (CFSAN044572), and BA2880 (CFSAN044573) belonging to ST101, and isolated from 2 specimens (blood and catheter) on the same patient (Lomonaco et al., 2018).

### Transfer experiments

*bla*_NDM-1_ or *bla*_OXA-48-_plasmids from the above 10 *K. pneumoniae* isolates were transferred to an *E. coli* J53 recipient strain. Conjugal transfer of carbapenem resistance between the *K. pneumoniae* isolates and *E. coli* J53, an azide-resistant recipient strain susceptible to all antibiotics, was performed as previously described (Lascols et al., 2008). Briefly, all isolates were grown to logarithmic phase in Brain Heart Infusion broth (BHI; ThermoFisher Scientific, MA, USA), and 2 ml of the donor and the recipient strain suspensions were mixed in flasks and incubated at 37°C for 40 min without shaking. Transconjugant selection was performed on Mueller-Hinton (ThermoFisher Scientific, MA, USA) plates containing sodium azide (100 μg/ml; ThermoFisher Scientific, MA, USA) and cefotaxime (16 μg/ml) or ticarcillin (100 μg/ml; TOKU-E, WA, USA) for transfer of *bla*_NDM-1_ and/or *bla*_OXA-48_-plasmids, respectively. Plates were incubated at 37°C and inspected at 24 and 48 h for bacterial growth.

### Antimicrobial susceptibility testing

Nine *E. coli* transconjugants were obtained: Ec_pBU19801_NDM, Ec_pMS84_NDM, Ec_pBL12125_NDM, Ec_pBL12456_NDM, Ec_pBA3783_NDM, Ec_pBL13802_NDM&OXA-48, Ec_pBA2664_OXA-48, Ec_pBL8800_OXA-48, and Ec_pBA2880_OXA-48. AST was carried out according to Clinical and Laboratory Standards Institute (CLSI) guidelines (CLSI, 2018; CLSI, 2023) and results interpreted using CLSI and European Committee on Antimicrobial Susceptibility Testing (EUCAST) breakpoints (EUCAST, 2023) for the above *E. coli* transconjugants.

### DNA extraction

Different DNA extraction methods were used for total genomic and plasmid DNA purification. Total genomic DNA was extracted using the QIAGEN DNA Mini Kit (QIAGEN, Valencia, CA, USA) per the manufacturer’s instructions from a 10 μl loopful of cells harvested from isolated colonies grown overnight.

Plasmid DNA was extracted from 100 ml overnight culture using the QIAGEN Plasmid Midi Kit (QIAGEN, Valencia, CA, USA) per the manufacturer’s instructions. For the *K. pneumoniae* isolates, the QIAGEN low-copy plasmid protocol was modified: the volumes of P1, P2, and P3 buffers were doubled (8 ml instead of 4 ml). DNA concentrations and purity were measured using a Qubit 3.0 fluorometer (ThermoFisher Scientific, MA, USA) with a dsDNA Broad Range Assay Kit and Nanodrop (ThermoFisher Scientific, MA, USA), respectively. DNA quality was assessed by measuring absorbency ratios at 260 nm/280 nm and at 260 nm/230 nm to ensure that the manufacturer’s recommended ratios of 1.8 and 2.0–2.2, respectively, were met prior to sequencing library preparation.

### Illumina MiSeq library preparation and sequencing

Libraries for Illumina MiSeq sequencing were prepared from total genomic DNA of each original isolate, and each *E. coli* transconjugant using the Nextera XT library prep kit (Illumina, San Diego, CA, USA), following the manufacturer’s instructions. DNA libraries were sequenced on a MiSeq instrument using 2×250 bp paired-end MiSeq Reagent Kit v2 (Illumina, San Diego, CA, USA) chemistry.

### Multiplexed nanopore library preparation and sequencing

MinION sequencing libraries were prepared from plasmid DNA preparations from both *K. pneumoniae* isolates and *E. coli* transconjugants. The SQK-RBK004 rapid barcoding kit was used according to the manufacturer’s protocol (Oxford Nanopore Technologies (ONT), Oxford, UK). The constructed library was loaded into a R9.4 (FLO-MIN106) Flow Cell on a MinION device and run with the High Accuracy BaseCaller script of MinKNOW1.5.12 for 72 h. The long reads sequencing data were stored on the MinIT, and accessible in real-time during the run.

### Generation of assembly sequences

Whole genome *de novo* assemblies obtained from SPAdes Genome Assembler (Bankevich et al., 2012) were used for analyses. Plasmid *de novo* assemblies were obtained from four genome assemblers: Miniasm, WTDBG2, Canu, and Flye (Koren et al., 2017; Kolmogorov et al., 2019; Ruan and Li, 2020). Assemblers were run with default parameters, resulting in a genome size of 5.4 M as expected for *K. pneumoniae* isolates. Hybrid assemblies from raw MinION reads were generated first by aligning MiSeq reads to the corresponding *de novo* assembly using minimap2 (Li, 2018). These alignments and *de novo* assembly were used as input to the Pilon utility, generating the error-corrected, hybrid assembly (Walker et al., 2014). All plasmid sequences for *K. pneumoniae* isolates were uploaded to the publicly available NCBI BioProject PRJNA946140.

### NGS-based prediction of antimicrobial resistance and plasmid and replicon-type analysis

Once NGS data are generated, AMR analysis tools were used for fully characterizing the AMR gene content and determining the plasmid types. Some software tools (e.g., ResFinder /PlasmidFinder/pMLST) rely on regular updates and curation (Bortolaia et al., 2020; Carattoli and Hasman, 2020) to accurately identify known and newly emerged ARGs ARGs/plasmid types across diverse bacterial species. To generate a comprehensive genotypic AMR profile from long-read sequences, and perform plasmid-typing, three publicly available web-based tools developed by the Center for Genomic Epidemiology (CGE): ResFinder, PlasmidFinder and pMLST were used.

ResFinder 4.0^1^ was used to independently identify acquired AMR determinants and determine NGS-based predicted phenotypes (Bortolaia et al., 2020). Full-length AMR determinants sharing more than 98% similarity and 100% coverage with the genes of interest found in the ResFinder databases were considered “present,” and included in the organism’s NGS-based AMR profile. In case of discrepant findings, the presence/absence of an AMR gene was determined manually by conducting BLAST analysis. Each gene’s presence was interpreted as either resistant (R) or not resistant (NR) by ResFinder 4.0. All NGS-predicted phenotypes were compared with conventional BMD results for *E. coli* transconjugants.

Plasmid incompatibility (Inc) groups were assessed using PlasmidFinder 2.0^2^ from CGE with default settings. Briefly, PlasmidFinder uses a curated database of known plasmid replicons for identifying plasmid incompatibility groups in *Enterobacteriaceae* whole genome sequences (Carattoli and Hasman, 2020). The software assigns plasmids for identification of lineages and suggests possible reference plasmids for additional analysis.

pMLST analysis was carried ou t with pMLST 2.0,^3^ which is a web-based tool for bacterial typing of MDR *Enterobacteriaceae* for rapid detection of known plasmid types (Carattoli and Hasman, 2020).

Those web-based tools (ResFinder4.0, PlasmidFinder2.0 and pMLST2.0) were used for determining AMR profiles, plasmid Inc. groups and types for each long-read sequence from the *K. pneumoniae* isolates and their corresponding *E. coli* transconjugants.

### Comparative analysis of plasmid structure in *E. coli* transconjugants.

The analysis of the structure of the *bla*_NDM-1_ plasmids transferred to *E. coli* was performed using an intuitive interface for rapid annotation of plasmid sequences that does not require bioinformatics expertise and provide detailed easy-to understand diagrams (Partridge and Tsafnat, 2018).

## Results

All NDM-producers carried at least two MDR plasmids, one with *bla*_NDM-1_ and one carrying one or more genes encoding other ß-lactamases, either a non-ESBL (*bla*_TEM-1B_) and/or an ESBL (*bla*_CTX-M-15_); except isolate MS84, which did not carry any *bla*_ESBL_-plasmid (Table 1). All OXA-48-producers carried two to three plasmids, one with *bla*_OXA-48,_ one with *bla*_TEM-1B_, and/or an additional plasmid containing ARGs ARGs such as those encoding aminoglycoside and/or rifampicin resistance (Table 1). Plasmids were transferred to *E. coli* J53 via conjugation to isolate *bla*_NDM-1_- and *bla*_OXA-48-_plasmids for sequencing and analysis. Overall, a total of nine *E. coli* transconjugants harboring either *bla*_NDM-1_ (*n* = 6) or *bla*_OXA-48_ (*n* = 3) plasmids were obtained from the 10 *K. pneumoniae* isolates. No transconjugant was obtained for BL849. For the two *bla*_NDM-1/OXA-48_ positive isolates (BA3783 and BL13802): only *bla*_NDM-1-_plasmid was successfully transferred from BA3783, while both *bla*_NDM-1_ and *bla*_OXA-48-_plasmids were successfully transferred from BL13802 (Table 1).

**Table 1:** List of MDR plasmids identified from Nanopore hybrid plasmid assemblies from 10 MDR *K. pneumoniae* clinical isolates and their respective *E. coli* transconjugants.

Isolate name	Plasmid name	Size (kb)	Plasmid replicon/Type	Plasmid typing
BL849	pBL849_NDM	277	IncFIB(pNDM-Mar); IncHI1B (pNDM-MAR)	-
	pBL849_ESBL	89	IncFIB(pQil)	-
BU19801	pBU19801_NDM	116	IncFIB(pQil); IncFII(K)	FIIK_2
	pBU19801_ESBL	181	IncFIB(K)*; IncFII(K)	FIIK_7
	*Ec_pBU19801_NDM*	116	IncFIB(pQil); IncFII(K)	FIIK_2
MS84	pMS84_NDM	142	IncC	ST1
	*Ec_pMS84_NDM*	142	IncC	ST1
BL12125	pBL12125_NDM	177	IncC	ST3
	pBL12125_ESBL	128	IncFII(K); repB(R1701)	-
	*Ec_pBL12125_NDM*	177	IncC	ST3
BL12456	pBL12456_NDM	177	IncC	ST3
	pBL12456_ESBL	128	IncFII(K); repB(R1701)	-
	*Ec_pBL12456_NDM*	177	IncC	ST3
BA3783	pBA3783_NDM	116	IncFIB(pQil); IncFII(K)	FIIK_2
	pBA3783_ESBL	135	FIA(pBK30683)	FIA_26
	*Ec_pBA3783_NDM + OXA-48*	116	IncFIB(pQil); IncFII(K)	FIIK_2
		62	IncL	-
BL13802	pBL13802_NDM	130	IncFII(pKPX1)	FIIK_1
	pBL13802_ESBL	135	IncFIB(K)	-
	pBL13802_OXA-48	62	IncL	-
	*Ec_pBL13802_NDM*	130	IncFII(pKPX1)	FIIK_1
	*Ec_pBL13802_OXA-48*	62	IncL	-
BA2664	pBA2664_OXA-48	62	IncL	-
	pBA2664_AAC	69	IncC	ST1
	*Ec_pBA2664_OXA-48*	62	IncL	-
BL8800	pBL8800_OXA-48	62	IncL	-
	pBL8800_ESBL	114	IncFIB(K); IncFII(pKP91)	FIIK_9
	pBL8800_AAC	121	IncFII(pKPX1)	-
	*Ec_pBL8800_OXA-48*	*62*	IncL	-
BABL2880	pBABL2880_OXA-48	62	IncL	
	pBABL2880_ESBL	114	IncFIB(K)*; IncFII(pKP91)	FIIK_9
	pBABL2880_AAC	121	IncFII(pKPX1)	-
	*Ec_pBABL2880_OXA-48*	62	IncL	-

The *E. coli* transconjugants (italics) harboring blaNDM-1-plasmid (*n* = 6), blaOXA-48-plasmid (*n* = 3) or both plasmids (*n* = 1) are highlighted in blue, green and brown, respectively.

### Comparison of assembly tools for generating hybrid plasmid assemblies

**Figure 1:**
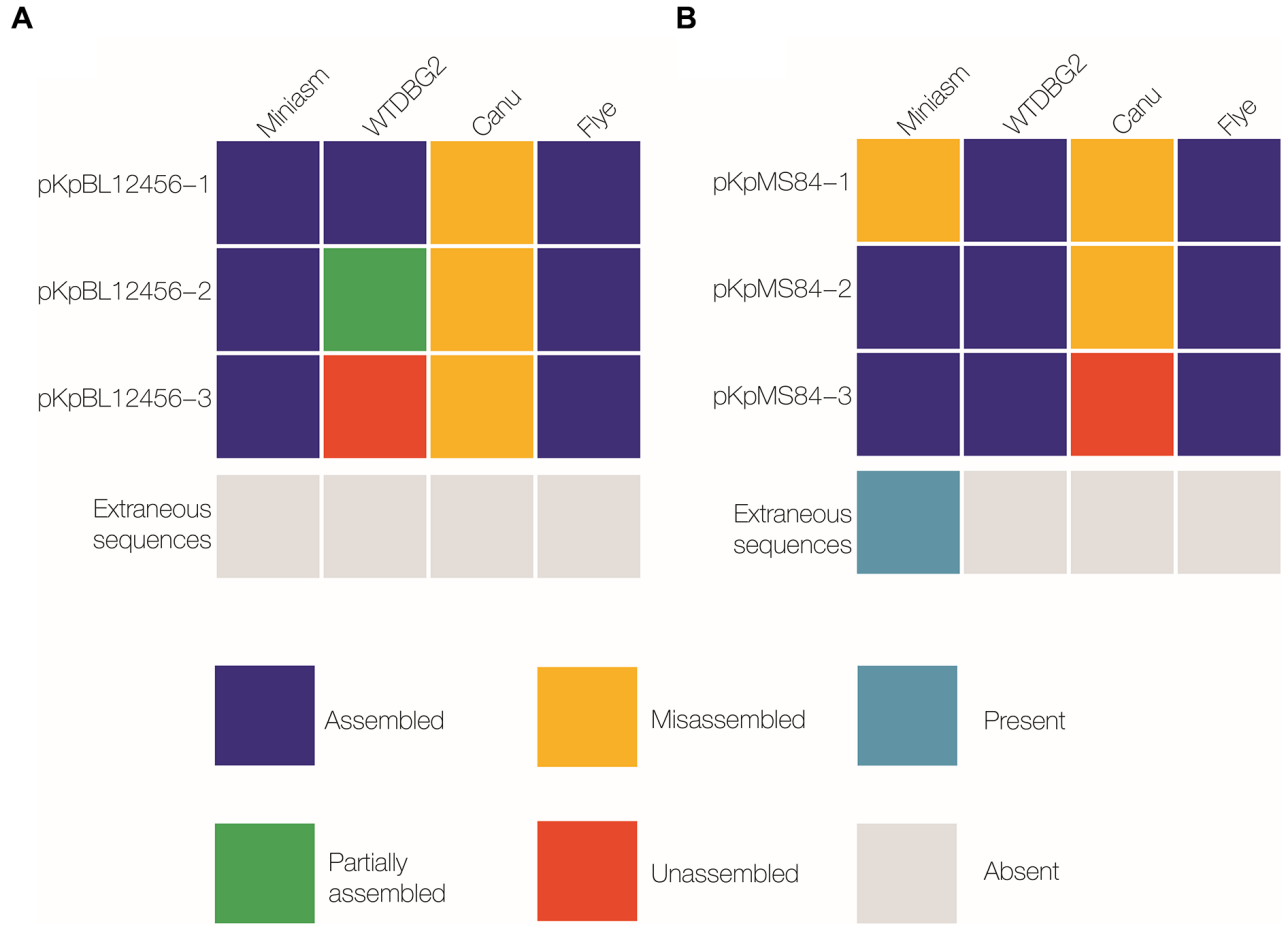
Comparison of plasmid-sequence assembly by Miniasm, WTDBG2, Canu, and Flye. Three plasmids were isolated from each of two *K. pneumoniae* strains: BL12456 (pKpBL12456-1, −2 and −3; **A**), and MS84 (pKpMS84-1, −2 and −3; **B**). Assembled was considered a complete sequence with the same nucleotide order as the reference sequence (navy); partially assembled represented a sequence that was assembled, but not as a single contig (green); misassembled included a sequence that was assembled, but the bases were in a different order than the reference sequence, or a sequence assembled multiple times resulting in an incomplete assembly (yellow); unassembled refers to a sequence that was not present in the reference assembly (orange).

Multiple assemblers can be used for whole genome DNA assembly, but we assessed which assembler would perform best on plasmids by comparing the most common assemblers: Miniasm, WTDBG2, Canu, and Flye. Overall, six PacBio plasmid sequences were used for reference: three originating from BL12456 (BL12456_pNDM, BL12456_pESBL; Table 1), and a non-AMR plasmid, and three plasmids from MS84 (MS84_pNDM; Table 1), and two non-AMR plasmids. Canu could not accurately assemble any of the six plasmid sequences. WTDBG2 correctly assembled four plasmid sequences, and Miniasm assembled five out of six plasmid sequences. Flye accurately assembled all six plasmid sequences, thus being the assembler that produced the most accurate and reliable hybrid assemblies. Therefore, Flye was used to generate assemblies for our collection of isolates (Figure 1). This finding is consistent with a new pipeline WeFaceNano for complete ONT sequence assembly and detection of AMR in plasmids (Heikema et al., 2021).

### Distribution and identification of plasmid-encoded AMR determinants by antimicrobial classes using ResFinder

ResFinder identified a total of 34 plasmid-mediated AMR determinants from hybrid assemblies of the 10 MDR *K. pneumoniae* isolates. Out of the 34 plasmid-mediated AMR determinants identified, the frequencies by antimicrobial agent classes were: β-lactams (23%, *n* = 8); aminoglycosides (35%, *n* = 12; trimethoprim and macrolides, 9% each, *n* = 3); 6% (fluoroquinolones, sulfamethoxazole, chloramphenicol, 6% each, *n* = 2). For tetracycline and rifampicin, the frequency was 3% for each (*n* = 1; Supplementary Figure S1). Excluding the 2 pairs of similar *K. pneumoniae* isolates, *bla*_NDM-1_, *bla*_ESBL_, and *bla*_OXA-48_-plasmids were identified in 6, 6 and 4 out of a total of 8 *K. pneumoniae* isolates, respectively. Single *bla*_NDM-1_ and *bla*_OXA-48_*-*plasmids were identified in two *K. pneumoniae* isolates (BA3783 and BL13802).

A total of eight different β-lactamase genes, encoding enzymes belonging to four classes, were identified: *bla*_NDM-1_, *bla*_OXA-10,_ *bla*_CMY-6_, and *bla*_CMY-16_ on NDM plasmids; *bla*_CTX-M-15,_ *bla*_OXA-1,_ and *bla*_TEM-1_, on *bla*_ESBL_-plasmids, and *bla*_OXA-48-_plasmids (Table 2). A total of 12 different plasmid-associated aminoglycoside resistance genes were identified: *aac(6′)-Ib, aph(3″)-Ib*, *aph(6)-Id*, *aadA1/A2*; *aph(3′)-VIb*, *rmtC/F*, and *armA* on *bla*_NDM-1-_plasmids, and *aac(3)-IIa/d*, *aac(6′)-Ib-cr*, *aph(3′)-Ia*, *aph(3″)-Ib*, and *aph6-Id* on *bla*_ESBL_*-*plasmids (Table 2). A total of two plasmid-mediated *qnr* genes were identified in four *K. pneumoniae* isolates (*qnrB1*, *n* = 3; *qnrS1*, *n* = 1) on *bla*_ESBL-_plasmids and *aac(6′)-Ib-cr* was identified in 6 *K. pneumoniae* isolates on *bla*_ESBL_-plasmids as well. Three variants of the *dfrA* gene (*dfrA1*, *dfrA12*, and *dfrA14*), which confer resistance to trimethoprim, and two variants of the *sul* gene (*sul1* and *sul2*), that confer resistance to sulfamethoxazole, were detected on *bla*_ESBL_*-*plasmids. In five *K. pneumoniae* isolates, *dfrA* and *sul* genes were found together on *bla*_ESBL_-plasmids: *dfrA1/sul1*, *dfrA14/sul2*, *dfrA14/sul1/sul2*, and *dfrA12/sul1*, but *sul1* alone was also identified on the *bla*_NDM-1_-plasmid in six *K. pneumoniae* isolates. The tetracycline resistance determinant *tetA* was identified on *bla*_ESBL_-plasmids from two *K. pneumoniae* isolates; a third *K. pneumoniae* isolate possessed a *tetA* gene with 99.92% similarity to the reference sequence (Genbank accession number: AJ517790) on *bla*_ESBL_*-*plasmid. The *arr2* gene conferring resistance to rifampicin, was identified on *bla*_NDM-1_-plasmids in six *K. pneumoniae* isolates. Macrolide resistance conferred by determinants such as *mphE* and *msrE* genes were identified on *bla*_NDM-1_-plasmids in three and four *K. pneumoniae* isolates, respectively. *mphA* was identified on *bla*_ESBL_-plasmid in one *K. pneumoniae* isolate. For chloramphenicol, *cmlA5* was identified on *bla*_NDM-1_*-*plasmids of two *K. pneumoniae* isolates. ESBL plasmids carried a *catB4* gene, which was subsequently found to be a truncated *catB3* gene in six *K. pneumoniae* isolates, and *catA1* was present in one *K. pneumoniae* isolate (data not shown).

**Table 2:** Antimicrobial resistance determinants identified from Nanopore hybrid plasmid assemblies from 10 MDR *K. pneumoniae* clinical isolates.

	pBL849_NDM	pBL849_ESBL	pBU19801_NDM	pBU19801_ESBL	pMS84_NDM	pBL12125/BL12456_NDM	pBL12125/BL12456_ESBL	pBA3783_NDM	pBA3783_ESBL	pBA3783_OXA-48	pBL13802_NDM	pBL13802_ESBL	pBL13802_OXA-48	pBA2664_OXA-48	pBA2664_AAC	pBL8800/BABL2880_OXA-48	pBL8800/BABL2880_ESBL	pBL8800/BABL2880_AAC
β-LACTAMS																		
*bla*_CMY-6/16_					6^1^	16												
*bla*_CTX-M-15_		x		x			x		x			x						
*bla*_NDM-1_	x		x		x	x		x			x							
*bla*_OXA-1/10/48_	10	1		1		10	1		1	48			48	48		48	1	
*bla*_SHV-1, −11, −28_																		
*bla*_TEM-1B_		x		x			x										x	
AMINOGLYCOSIDES																		
*aac(3)-Iia*		x		x			x					x			d		a	
*aac(6′)-Ib3*			x		x			x							x			
*aac(6′)-Ib-cr*		x		x			x		x			x					x	x
*aacA4*																		
*aadA1/A2*	A1					A1						A2						
*aph(3′)-Ia*												x						
*aph(3″)-VIb = aphA6*	x																	
*aph(3″)-Ib = strA*		x	x	x			x	x									x	
*aph(6)-Id = strB*		x	x	x			x	x										
*armA*	x		x					x										
*rmtC/F*					C						F							F
FLUOROQUINOLONES																		
*qnrB1/S1*		S1					B1		B1									
TMP/SXT																		
*dfrA1/12/14*		1		14			14		14			A12						
*sul1/2*	1	1	1	2	1	1	2	1				1			1		2	
TETRACYCLINES																		
*tetA*		x					x											
CHLORAMPHENICOL																		
*catA1*		A1																
*cmlA1*	x					x												
MACROLIDES																		
*mphA/E*			E			E		E				A						
*msrE*	x		x			x		x										
RIFAMPICIN																		
*arr2*	x					x					x							x
*Number of ARGs*	9	12	9	9	6	9	11	9	5	1	4	7	1	1	3	1	6	3

1 An “x” indicates the presence of a gene as determined by ResFinder 4.0.

2 The number indicates the variant identified for that gene (e.g., 10 refers to blaOXA-10).

3 The ARGs identified on blaNDM-1-, blaESBL-, and blaOXA-48-plasmids are highlighted in pink, blue, or green, respectively. The ARGs identified at 95–99.9% are highlighted in light pink, blue or green.

4 A dark pink or blue cell or allele designation indicates 100% identity. A threshold of 98% identity along with a full gene length were used to determine the presence of a gene.

### Analysis of NGS-based genotypic AMR profiles and phenotypes from plasmids

NGS-based AMR profiles were generated from the hybrid assemblies obtained from plasmid preps from both the original *K. pneumoniae* isolates, and the *E. coli* transconjugants. The plasmids isolated from *K. pneumoniae* isolates are noted with a p in front of the isolate designation (e.g., pMS84) while the plasmids isolated from the *E. coli* transconjugants are noted with Ec before the isolate name (e.g., Ec_pMS84_NDM). The presence of both *bla*_NDM-1_ or *bla*_OXA-48_ and *bla*_ESBL_ plasmids in the original *K. pneumoniae* isolates could not explain the contribution of each plasmid in NGS-based AMR profiles, while the NGS-based AMR profiles obtained from *E. coli* transconjugants gave insights on the contribution of ARGs ARGs present on *bla*_NDM-1_ or *bla*_OXA-48_ plasmids. All detected ARGs ARGs, NGS-predicted AST results for *K. pneumoniae* isolates, and *E. coli* transconjugants, and AST results for *E. coli* transconjugants are summarized in Supplementary Table S2 (β-lactams), Supplementary Table S3 (aminoglycosides), Supplementary Table S4 (fluoroquinolones), and Supplementary Table S5 (trimethoprim/sulfamethoxazole, tetracyclines, and chloramphenicol).

#### Resistance to β-lactams

All five *K. pneumoniae* isolates (BL849, BU19801, MS84, BL12125/456) carrying *bla*_NDM-1_ were resistant to all classes of β-lactam antimicrobials. Among these isolates, three (BL849, BL12125, and BL12456) carried up to five β-lactamase genes: one *bla*_CTX-M-15_ ESBL gene, one *bla*_OXA-1_ along with *bla*_TEM-1B_ on the *bla*_ESBL_-plasmid, and *bla*_OXA-10_ on the *bla*_NDM-1_-plasmid, including two isolates that were found to carry the *bla*_CMY-16_ AmpC β-lactamase gene on the *bla_NDM-1_*-plasmid. BU19801 carried three β-lactamase genes (*bla*_CTX-M-15,_ *bla*_OXA-1,_ and *bla*_TEM-1B_) on the *bla*_ESBL_-plasmid while MS84 carried in addition to *bla*_NDM-1_ only one gene variant of AmpC β-lactamase (*bla*_CMY-6_) on the *bla*_NDM-1_-plasmid. Since most β-lactamase genes were located on the *bla*_ESBL_-plasmid, when *bla*_NDM-1_ was transferred by itself, the *E. coli* transconjugants were susceptible to aztreonam which agrees with the definition of NDM-1 producers except for BL12125 and BL12456. When additional β-lactamase genes were transferred, such as *bla*_CMY-16_ and *bla*_OXA-1_ (BL12125 and BL12456), the *E. coli* transconjugant isolates were resistant to all β-lactams tested by both the NGS-based approach and confirmed by BMD.

For most *bla*_NDM-1_-positive *E. coli* transconjugants, the NGS-based AMR profiles were in concordance with conventional AST results, and consistent with the characteristics of NDM-1 producers [example: NDM-1 producers hydrolyze all ß-lactams except aztreonam (ATM)]. However, the absence of *bla*_CTX-M-15_ was interpreted by ResFinder as not resistant to ceftriaxone (CRO) and ATM for five *bla*_NDM-1_-positive (Ec_pBU19801_NDM, Ec_pMS84_NDM, Ec_pBL12125/456_NDM, Ec_pBA3783_NDM, and Ec_pBL13802_NDM) and two *bla*_OXA-48_-positive *E. coli* transconjugants (Ec_pBA2664_OXA-48, Ec_pBL8800/pBABL2880_OXA-48) respectively, and one *K. pneumoniae* isolate (MS84). MICs for CRO and ATM were ≥32 μg/ml for those *K. pneumoniae/E. coli* isolates which is interpreted as resistant (≥4 μg/ml) by CLSI (2023), therefore those NGS-based results did not match BMD results (Supplementary Table S1).

Among the three *bla*_OXA-48_-positive *K. pneumoniae* isolates, two (BL8800 and BA2880) carried both *bla*_TEM-1_, and *bla*_OXA-1_ located on a *bla*_ESBL_-plasmid and were resistant to penicillins [ampicillin (AMP), amoxicillin-clavulanic acid (AMC), ampicillin-sulbactam (SAM), piperacillin-tazobactam (TZP)], cephalosporins [cefazolin (CZ), cefotaxime (CTX), ceftazidime (CAZ), CRO, cefepime (FEP)], and ATM and yet were susceptible to doripenem (DOR), meropenem (MEM) and non-susceptible to imipenem (IMP). One *K. pneumoniae* isolate (BA2664) did not carry any *bla*_ESBL_-plasmid and was resistant to all ß-lactams except CAZ and ATM.

The three *E. coli* transconjugant isolates that carried the *bla*_OXA-48_ plasmid were also resistant to the first generation of β-lactams, but not to the more recent generations of cephalosporins (FOX, CTX, CAZ, CRO, and FEP) and carbapenems (IPM, MEM, ERT) except DOR. This susceptibility profile is consistent with the observation that OXA-48 producers hydrolyze carbapenems at a low level (Poirel et al., 2012). However, in two instances (Ec_pBL8800/BABL2880_OXA-48), the presence of *bla*_OXA-48_ was interpreted by ResFinder as resistant for IPM and MER, which does not agree with MICs for IPM and MEM of 1 and 2 μg/ml (susceptible), respectively (Supplementary Table S1).

#### Resistance to aminoglycosides

A total of 11 different genes responsible for high-level aminoglycoside resistance: (i) N-acetyltransferases (AAC): *aac(3)-IIa*, *aac(6′)-Ib-cr*; (ii) aminoglycoside-O-nucleotidyltransferases (ANT): *aadA1,* and *aadA2*; (iii) aminoglycoside-O-phosphotransferases (APH): *aph(3′)-Ia*, *aph(3″)-Ib*, *aph(6)-Id, aph(3′)-VIb; n* = 4; and (iv) 16S rRNA methylases (*armA, rmtC or rmtF*) were identified in *E. coli* transconjugants (Supplementary Table S2). All of the *E. coli* transconjugants carrying *bla*_NDM-1_-plasmids were resistant to AMK (MIC > 64 μg/ml), GM (MIC > 16 μg/ml), and TOB (MIC > 16 μg/ml) by ResFinder and confirmed by BMD.

Out of the six *bla*_NDM-1_-positive *E. coli* transconjugants, each of the *bla*_NDM-1_-plasmids carried at least one AMR gene conferring resistance to amikacin (AMK), gentamicin (GM) and tobramycin (TOB). Four carried *aac(6′)-Ib-cr* either solely (*n* = 2) or with *armA* (*n* = 2), *rmtC* (*n* = 1), or *rmtF* (*n* = 1). In addition to all the ARGs identified on the NDM plasmids, most *K. pneumoniae* isolates harbored additional ARGs conferring resistance to aminoglycosides such as *aac3-IIa* on the *bla*_ESBL_-plasmids except MS84 and BA3783. Ec_pBA2664_OXA-48 did not carry any aminoglycoside resistance gene except *aac3-IId* located on a non-*bla_ESBL_*-plasmid but was called resistant by ResFinder, which did not agree with MICs of susceptible of 8 μg/ml.

Other ARGs conferring resistance to STR (not tested by BMD) such as *aadA2* and *aph(3′)-Ia* were identified on the *bla*_ESBL_-plasmid (pBL13802) of a single *K. pneumoniae* isolate. The *aph(3″)-Ib* gene also called *strA* was identified on four *bla*_ESBL_-plasmids (pBL849_ESBL, pBU19801_ESBL, pBL12125/456_ESBL and pBL8800/BABL2880_ESBL) and two *bla*_NDM-1_-plasmid (pBU19801_NDM and pBA3783_NDM). *aph(6)-Id* also called *strB* was identified on the same above plasmids except for pBL8800/BABL2880_NDM for which *aph(6)-Id* was missing. Two *E. coli* transconjugants carried *aadA1* and two carried both *aph(3″)-Ib* and *aph(6)-Id.*

#### Resistance to fluoroquinolones

All nine *E. coli* transconjugants that were susceptible to CIP (MICs ≤0.25 μg/ml) did not carry any *aac(6′)-Ib-cr, qnrB,* or *qnrS*. However, ResFinder identified a truncated version of *aac(6′)-Ib-cr*-EF636461 (519 bp) in 4/9 *E. coli* transconjugants (Supplementary Table S3) which were categorized as resistant. This observation suggests that this truncated *aac(6′)-Ib-cr* may not confer resistance to fluoroquinolones and should be removed from the ResFinder database until confirmation.

#### Resistance to trimethoprim-sulfamethoxazole (cotrimoxazole), tetracyclines, and chloramphenicol

Modified dihydrofolate reductase (DHFR) and dihydropteroate synthetase (DHPS), enzymes encoded by *dfr* and *sul*, respectively, are involved in resistance to cotrimoxazole (SXT; trimethoprim + sulfamethoxazole; Ramirez and Tolmasky, 2010). The five *E. coli* transconjugants carrying *bla*_NDM-1_-plasmid that harbored *sul1* without any *dfr* genes likely explained their susceptibility to SXT. The absence of the *dfr/sul* genes on *bla*_NDM-1_-plasmid for four *E. coli* transconjugants was consistent with the observed susceptibility to SXT. However, Resfinder does not propose any interpretation categorization for SXT susceptibilities. The absence of the *tetA* gene on *bla*_NDM-1_-plasmid was consistent with the observed susceptibility to TET by AST. Two *E. coli* transconjugants, carrying *cmlA5* on the *bla*_NDM-1_ plasmid, were susceptible to CHL (MIC = 8 μg/ml) but categorized as resistant to CHL by Resfinder (Supplementary Table S4).

### Structure of MDR plasmids identified in *MDR Klebsiella pneumoniae clinical* isolates

The structures of each plasmid transferred *via* conjugation and identified in *E. coli* transconjugants were consistent with the plasmids identified in the *K. pneumoniae* study isolates (100% similarity). The *bla*_NDM-1_-plasmid was the only plasmid that was transferred for BA3783 while both *bla*_NDM-1_- and *bla*_OXA-48_-plasmids were transferred for BL13802. Lastly, no transconjugant was obtained for BL849. The incompatibility groups identified for (1) *bla*_NDM_-, (2) *bla*_ESBL_, and (3) *bla*_OXA-48_-plasmids were: (1) IncFIB, IncHI1B, IncFII, and IncC; (2) IncFIB; and IncFII; and (3) IncL, respectively. The pMLST types identified were IncC11 and IncC3 (*n* = 2), IncFII2 and IncFII1 among *bla*_NDM-1_-plasmids and were IncFIIK7, IncFIIK9, and IncFIA26 among *bla*_ESBL_-plasmids (Table 1).

#### NDM-producers (*n* = 7): BL849, BU19801, MS84, BL12125, BL12456, BA3783, BL13802

Among the seven *bla*_NDM-1_-positive *K. pneumoniae* isolates, the number of AMR determinants encoded on *bla*_NDM-1_- and *bla*_ESBL_-plasmids varied from 4 to 10, and 7–12 genes, respectively. Whole-plasmid hybrid sequencing identified seven *bla*_NDM-1_-plasmids of various sizes ranging from 117 to 277 kb: pBL849_NDM (277 kb), pBU19801_NDM (181 kb), pMS84_NDM (142 kb), pBL12125_NDM and pBL12456_NDM (177 kb), pBA3783_NDM (117 kb), and pBL13802_NDM (130 kb). Among the seven *bla*_NDM-1_-plasmids, IncC was the most common incompatibility group identified (*n* = 3), followed by IncFIB (*n* = 2), IncFII (*n* = 1), and IncFIB/IncHI1B (*n* = 1; Table 1).

**Figure 2:**
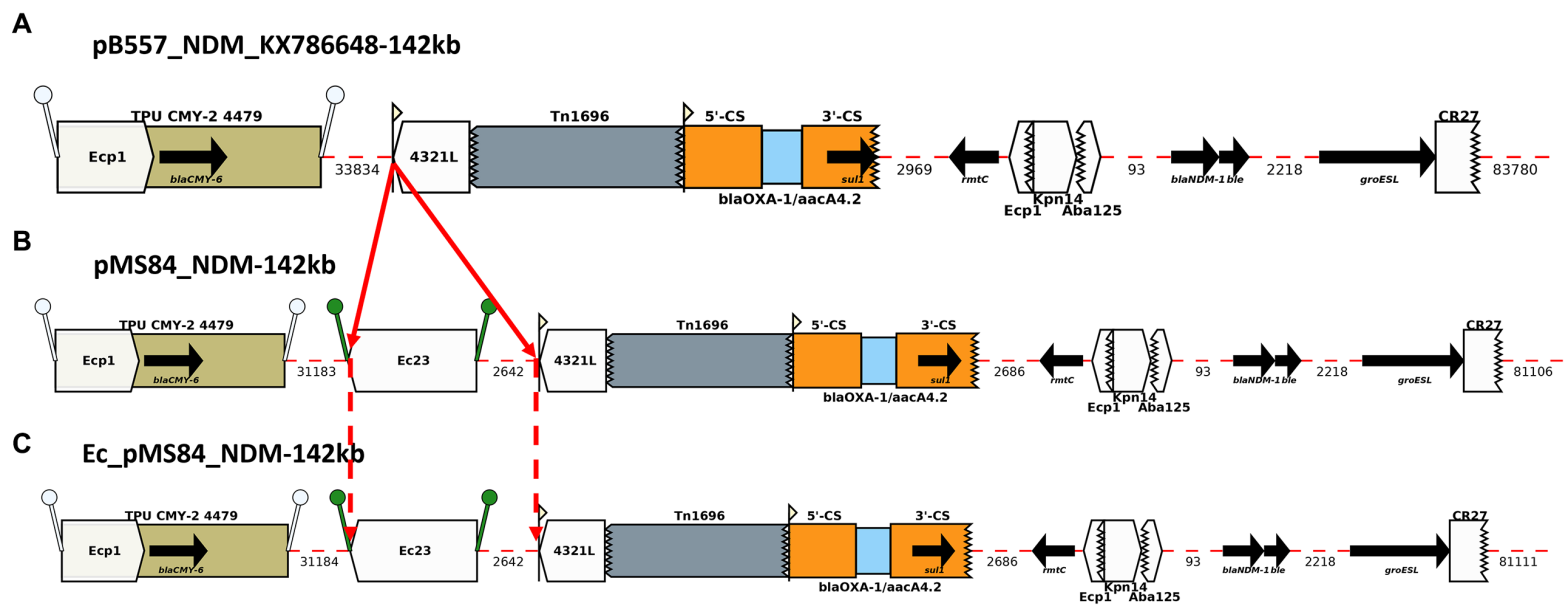
Annotation Diagram of **(A)** pB557-NDM_KX786648, **(B)** MS84_pNDM, and **(C)** Ec_MS84_pNDM using Galileo AMR platform. Gaps >50 (base pairs) bp are indicated by dashed red lines and the length in bp is given. Genes features (e.g., *bla*_CMY-6_, *sul1*, *rmtC*, *bla*_NDM-1_) are shown by arrows; gene cassettes (e.g., *bla*_OXA-1_/*aacA4.2*) by pale blue boxes; the CS of integrons as orange boxes; and IS (e.g., IS*Ec23*) as white block arrows labelled with the IS number/name and the pointed end indicating IR_R_. Unit transposons (e.g., Tn*1696*) are shown as boxes of different colors and their IR are shown as flags, with the flat side at the outer boundary of the transposon. Truncated features (e.g., Tn*1696*) are shown with a jagged edge on the truncated side(s). Direct repeats flanking ISs are shown as ‘lollipops’ of the same color ISEcp1 (white) and ISEc23(green). Here, an ISEc23 has been inserted in MS84_pNDM and Ec_MS84_pNDM compared to pB557-NDM-KX786648.

**Figure 3:**
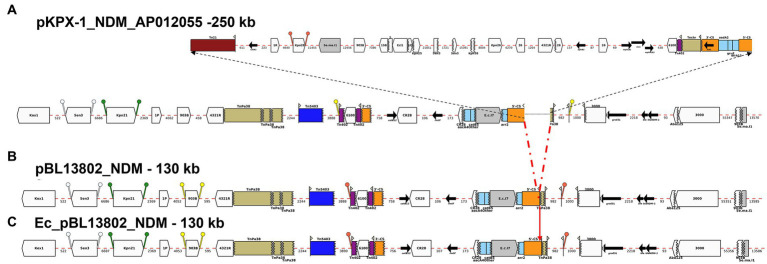
Annotation Diagram of **(A)** pKPX-1-NDM_AP012055, **(B)** BL13802_pNDM, and **(C)** Ec_BL13802_pNDM using Galileo AMR platform. Gaps >50 (base pairs) bp are indicated by dashed red lines and the length in bp is given. Genes features (e.g., *catB12*, *rmtF*, *bla*_NDM-1_) are shown by arrows; gene cassettes (*catB3*/*aacA4/arr2*) by pale blue boxes; the CS of integrons as orange boxes; and IS (e.g., IS*3000*) as white block arrows labelled with the IS number/name and the pointed end indicating IR_R_. Unit transposons are shown as boxes of different colors (e.g., Tn*5403*, blue, Tn*40*, dark purple) and their IR are shown as flags, with the flat side at the outer boundary of the transposon. Truncated features (e.g., 3’-CS) are shown with a jagged edge on the truncated side(s). Direct repeats flanking ISs are shown as ‘lollipops’ of the same color IS*Kpn21* (green) and IS903B (yellow). Here, a 46-kb region containing *aacA4*, *catB3*, and *arr2* cassettes is found in pKPX-1-A012055 upstream of *bla*_NDM-1_ both in BL13802_pNDM, and Ec_BL13802_pNDM.

BLAST analysis of the complete nucleotide sequences of MDR plasmids identified in this study found zero exact matches to previously reported MDR plasmids for five *K. pneumoniae* isolates: pBL849_NDM, pBU19801_NDM, pBL12125/456_NDM and pBA3783_NDM. However, pMS84_NDM and pBL13802_NDM each shared high similarity with two plasmids previously described, pB557-NDM-KX786648 (142 kb) and pKX-1-NDM-AP012055 (250 kb), respectively (Figures 2, 3).

pMS84_NDM shared 100% identity with pB557-NDM_KX786648 (GenBank accession number: KX786648), a *bla*_NDM-1_-plasmid isolated from an *Enterobacter cloacae* clinical isolate in China in 2016. An IS*Ec23* (5 kb) was inserted downstream the IS*Ecp1*/*bla*_CMY-6_ module and upstream of a Tn*1696* transposon in pMS84_NDM (Figure 2). The ARGs identified on this *bla*_NDM-1_-plasmid were: *bla*_CMY-6_, *sul1, rmtC*, and *bla*_NDM-1._

pBL13802_NDM was identical to part of the 250-kb total sequence (99.98% similarity) of plasmid pKPX-1-NDM-AP012055 (Genbank accession number: AP012055), a plasmid isolated from *K. pneumoniae* strain KPX-1, obtained from a Taiwanese patient with a hospitalization history in New Delhi (Huang et al., 2013). pBL13802_NDM lacked a 120-kb region from pKPX-1 plasmid. pBL13802_NDM contained a 22-kb AMR region, also found in pKPX-1-AP012055, composed of several gene cassettes, such as *catB3, aac(6′)-Ib*, *aacA4* and *arr2*, as well as a region containing *bla*_NDM-1_ (Figure 3).

**Figure 4:**
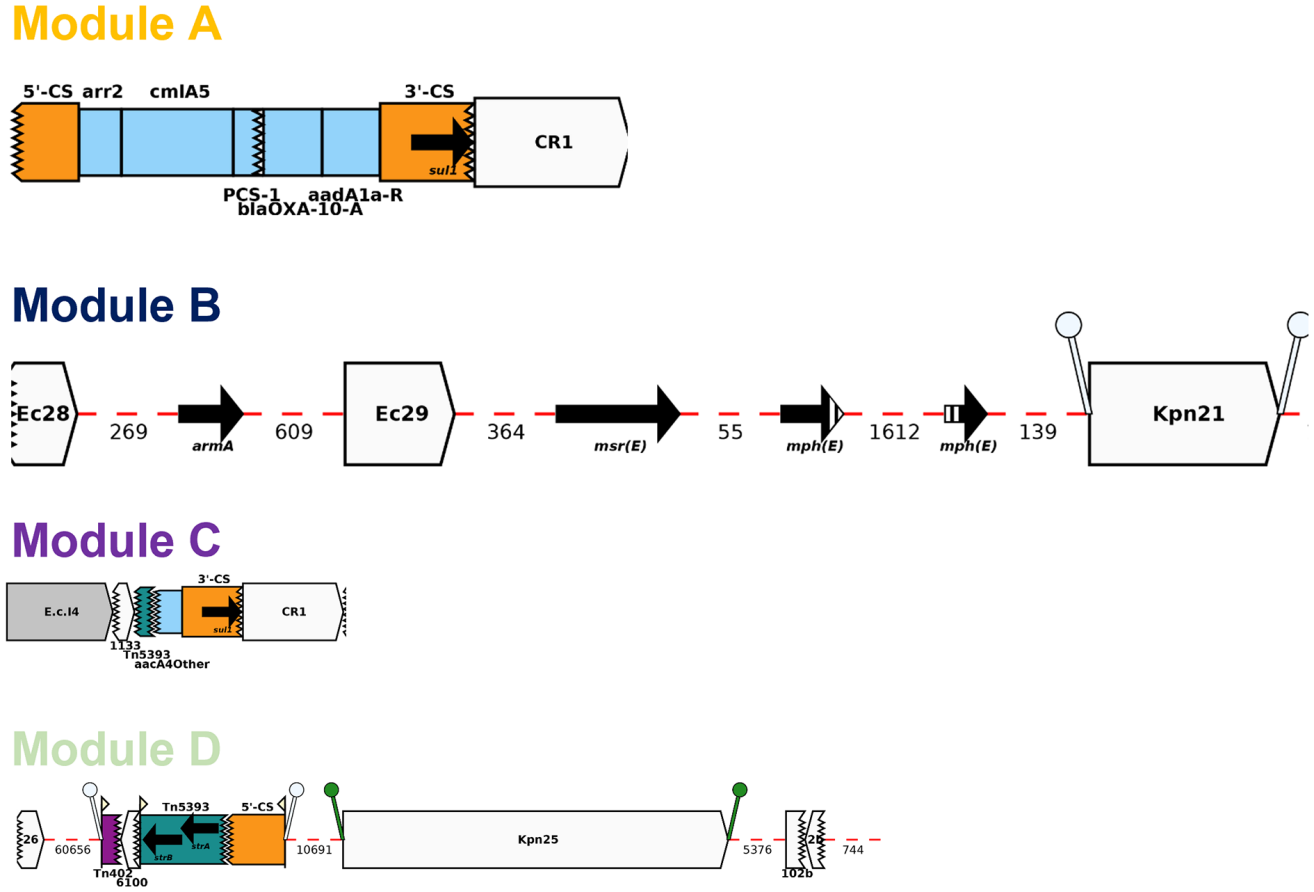
Representation of AMR modules A-D described in this study using Galileo AMR platform.

**Figure 5:**
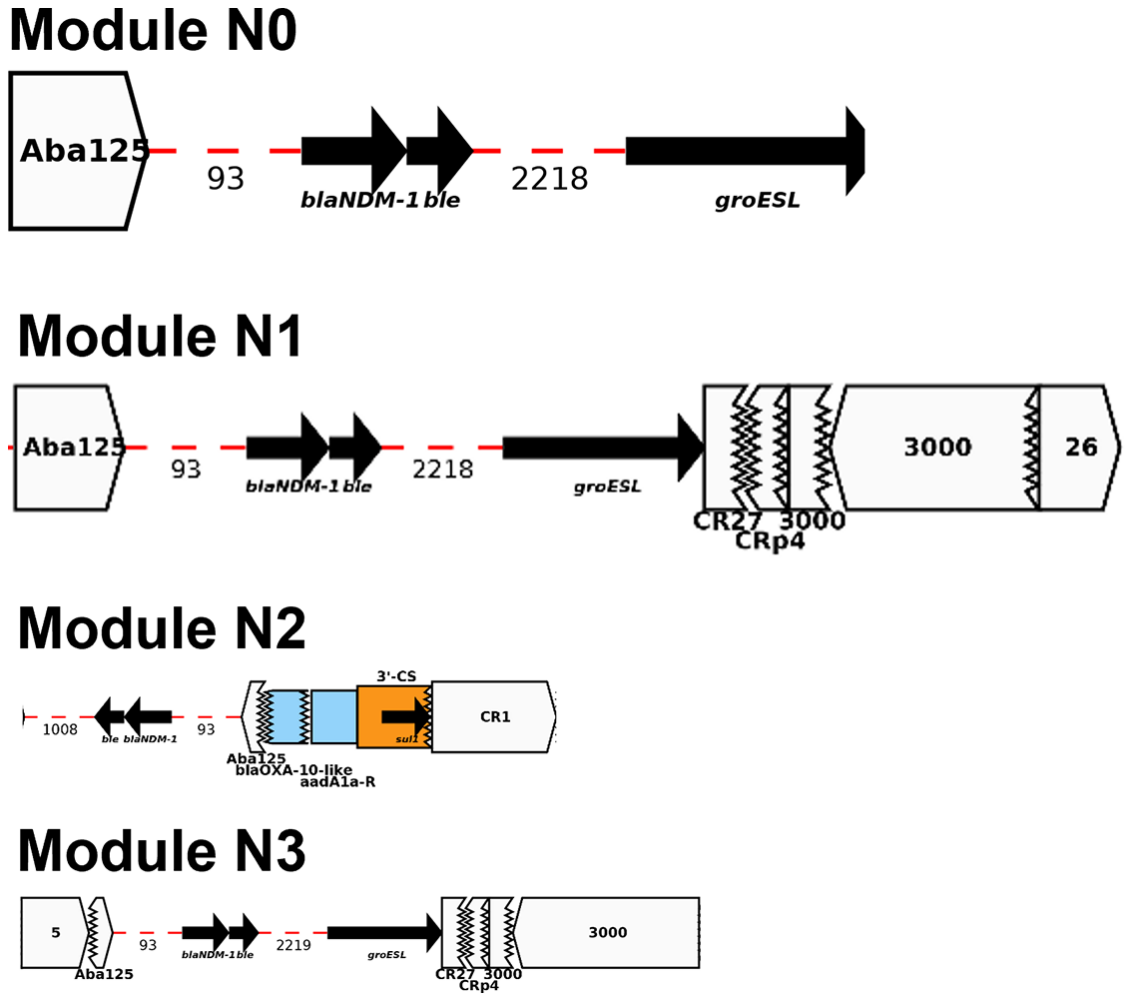
Representation of AMR modules N0-N3 described in this study using Galileo AMR platform.

BLAST analysis identified regions/AMR modules that were present in several of the study plasmids: Modules A-D and N0-N3. The AMR modules identified in this study are illustrated in Figures 4, 5, respectively. Module A is composed of a complete gene cassette of 5 ARGs: 3’-CS-[*arr2/cmlA5/bla*_OXA-10_*/aadA1/sul1*]-5’-CS. Module B has *armA, msrE*-like, and *mphE* (2 copies) genes, along with multiple insertion sequences (IS; IS*Ec28*, IS*Ec29*, and IS*Kpn21*). Module C contains two ARGs: a truncated *aac(6′)-Ib*-cr and *sul1*. Module D is composed of IS*Kpn25* along with two *strA/strB* ARGs (Figure 4).

Module N0 here is composed of *bla*_NDM-1_, *ble*_MBL_, and *gro*_ESL_ which is consistent with the backbone of a truncated ΔTn*125* transposon previously described in multiple *bla*_NDM-1_-plasmids (Dong et al., 2019). Prior studies showed that IS*Aba*125 mobilized “en bloc” both *bla*_NDM-1_ and bleomycin *ble*_MBL_ genes which originated from the same progenitor (Poirel et al., 2011). *bla*_NDM-1_ is mainly and widely spread by an IS*Aba125*-bounded composite transposon Tn*125* (Poirel et al., 2012). Module N1 is a section downstream of Module N with multiple truncated IS (IS*Cr27*/IS*CRp4*/IS*3000*) along with a complete sequence of IS*3000* and IS*26*. Module N2 is composed of Module N except *gro*_ESL_ is flanked upstream of IS*Aba125* by a set of three ARGs (e.g., *bla*_OXA-10_, *aadA1*, and *sul1*). Lastly, Module N3 is very similar with Module N1 with an addition of IS*5* upstream of IS*Aba125* (Figure 5).

**Figure 6:**
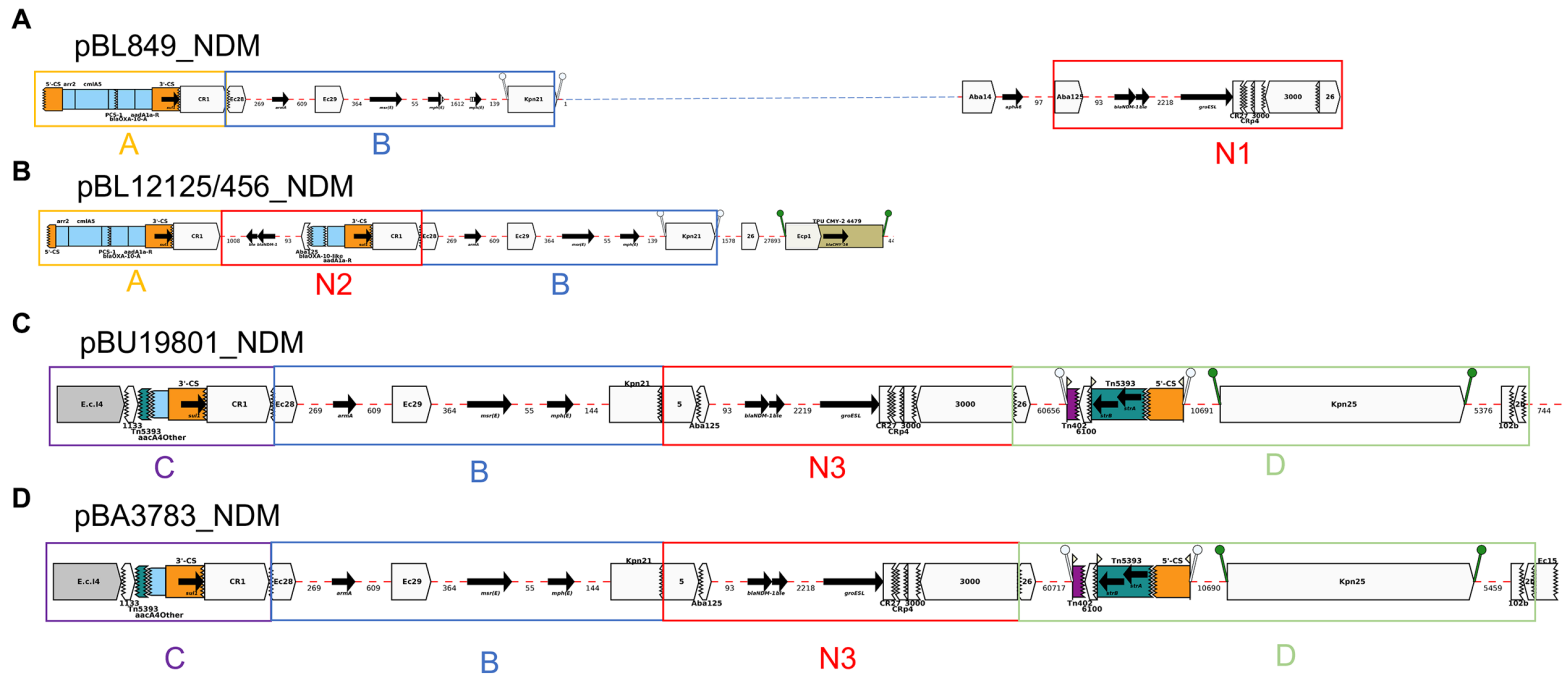
Annotation Diagrams of pNDM-plasmids **(A)** BL849_pNDM, **(B)** BL12125/456_pNDM, **(C)** BU19801_pNDM, and **(D)** BA3783_pNDM using Galileo AMR platform. Gaps >50 bp are indicated by dashed red lines and the length in bp is given. Genes features (e.g., *armA*, *sul1*, *bla*_NDM-1_) are shown by arrows; gene cassettes (*arr2/OXA-10/catB3*) by pale blue boxes; the CS of integrons as orange boxes; and IS (e.g., IS*3000*) as white block arrows labelled with the IS number/name and the pointed end indicating IR_R_. Unit transposons are shown as boxes of different colors (e.g., Tn*5393*, green) and their IR are shown as flags, with the flat side at the outer boundary of the transposon. Truncated features (e.g., 3’-CS) are shown with a jagged edge on the truncated side(s). Direct repeats flanking ISs are shown as ‘lollipops’ of the same color IS*Kpn21* (green). Modules A, B, C and D are represented by yellow, blue, purple, and light green boxes. Modules N1, N2 and N3 comprising *bla*_NDM-1_ are represented by red boxes.

Among the seven *bla*_NDM-1_-positive *K. pneumoniae*: pBL849_NDM and pBL12125/456_NDM, shared modules A and B and some variations of module N (N1, BL849 and N2, BL12125/456) while pBU19801_NDM and pBA3783_NDM, shared the exact same modules: B, C, D and N3 (Figure 6).

pBL849_NDM, and pBL12125/456_NDM shared a common set of seven ARGs (Table 2) including *arr-2, cmlA5, aadA1, sul1* as part of Module A, and *armA, msrE,* and *mphE* (2 copies for pBL849_NDM) as part of Module B. Also, pBL849_NDM and pBL12125/456_NDM carried different genes or IS around *bla*_NDM-1_ (Module N1 for pBL849_NDM and Module N2 for pBL12125/456_NDM; Figure 6).

pBU19801_NDM and pBA3783_NDM shared a common set of eight ARGs (Table 2) including truncated-*aac(6′)-Ib-cr* and *sul1* (Module C); *armA, msrE,* and *mphE* (Module B), and *aph(3″)-Ib* (or *strA*), *aph(6)-Id* (or *strB*; Module D) along with Module N3 (Figure 6). pBL12125/456 had the same AMR profiles for both *bla*_NDM-1_-(10 ARGs) and *bla*_ESBL_-(11 ARGs) plasmids. pBL12125/456_NDM also contained *bla*_CMY-16-like_ downstream of module B which was not present in pBL849_NDM. Conversely, pBL849_NDM also contained an additional AMR gene, *aph(3″)-VIb* (=*aph6*) upstream of Module N1 which was not present in pBL12125_NDM and pBL12456_NDM (Table 2; Figure 6).

Module A was shared by pBL849_NDM, pBL12125/456_NDM (*n* = 3) while module B was shared by pBL849_NDM, pBL12125/456_NDM, pBU19801_NDM, and pBA3783_NDM (*n* = 5). Modules C and D were shared by pBU19801_NDM and pBA3783_NDM (*n* = 2). Module N0 was shared by all five *K. pneumoniae* NDM-1 producers including modifications for pBL849_NDM (Module N1) pBL12125_NDM and pBL12456_NDM (Module N2), and pBU19801_NDM and pBA3783_NDM (Module N3; Figure 6).

#### OXA-48-producers (*n* = 5): BA3783, BL13802, BA2664, BL8800, and BA2880

**Figure 7:**
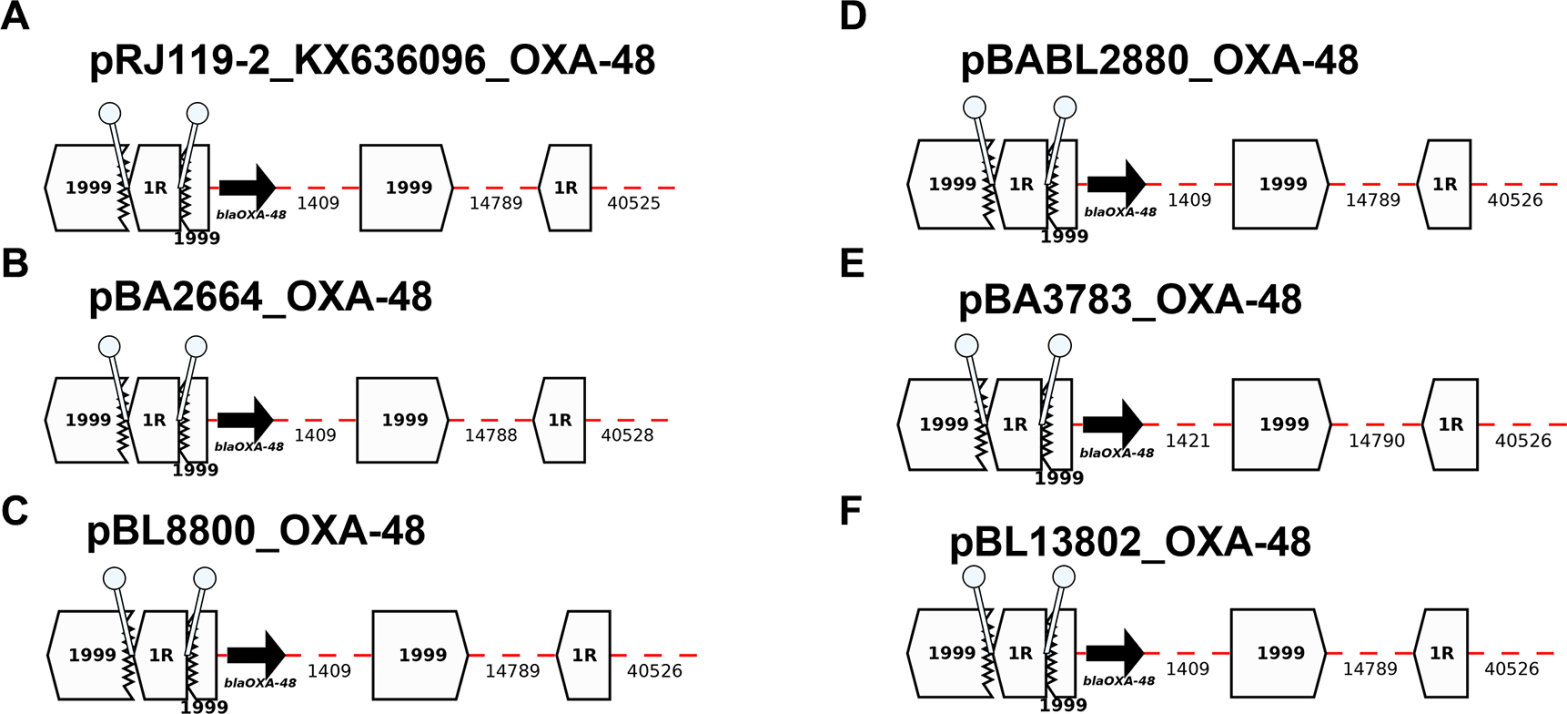
Annotation Diagrams of OXA-48-plasmids **(A)** pRJ119-1-OXA-48_KX636096, **(B)** pBA2664-NDM, **(C)** pBL8800_OXA-48, **(D)** pBABL2880_OXA-48, **(E)** pBA3783-OXA-48, and **(F)** pBL13802_OXA-48 using Galileo AMR platform. Gaps >50 bp are indicated by dashed red lines and the length in bp is given. Genes features (i.e., *bla*_OXA-48_) are shown by arrows, and IS (e.g., IS1999, IS1) as white block arrows labelled with the IS number/name and the pointed end indicating IR_R_. Truncated features (e.g., IS1999) are shown with a jagged edge on the truncated side(s). Direct repeats flanking ISs are shown as ‘lollipops’ of the same color IS1R (white). Here, the *bla*_OXA-48_ module found in all isolates are the same and identical to the module identified in pRJ119-2_KX636096.

All OXA-48 producers shared the same *bla*_OXA-48_-plasmid, which does not carry additional ARGs (Figure 7). All five *bla*_OXA-48_-plasmids were positive for IncL. BLAST analyses of the plasmid sequence revealed high similarity (99.99%) between the current 62-kb *bla*_OXA-48_-plasmid and pRJ-119-2, first *bla*_OXA-48_-positive plasmid isolated in China in 2016 (Figure 6). Tn*1999*, also named Tn*1999.1,* consists of two copies of the insertion sequence IS*1999* surrounding *bla*_OXA-48_: one copy inserted 26 bp upstream of *bla*_OXA-48_ and another copy downstream of *bla*_OXA-48_. In our sequences, the insertion of IS*1R* into IS*1999* upstream of *bla*_OXA-48_ indicates the presence of a Tn*1999.2* variant (Giani et al., 2012; Figure 7).

#### ESBL-plasmids: BL849, BU19801, MS84, BL12125, BL12456, BA3783, BL13802, BL8800, and BA2880

Among the 10 *bla*_NDM-1_ and/or *bla*_OXA-48_-positive *K. pneumoniae* isolates, eight *bla*_ESBL_-plasmids were characterized by hybrid sequencing. *K. pneumoniae* isolates pMS84 and pBA2664 did not carry *bla*_ESBL_-plasmids. Among the eight *bla*_ESBL_-plasmids, IncFIB was the most common incompatibility group identified with 100% identity (*n* = 2) followed by IncFII (*n* = 1). As far as incompatibility groups with an identity >96%, the IncFIB/IncFII combination was the most common (*n* = 3), followed by IncFII (*n* = 1) and FIA (*n* = 1; Table 1).

Five *bla*_ESBL_-plasmids identified in this study were highly comparable to some regions of previously described *bla*_ESBL_-plasmids. However, none of the prior *bla*_ESBL_-plasmids shared the exact same sequence structure with the *bla*_ESBL_-plasmids described herein. Also, 3/8 *bla*_ESBL_-plasmids shared identity in a region that did not contain ARGs: BU19801_pESBL (99.97%, a 180-kb region of *K. pneumoniae* E16KP0258 chromosome, Genbank accession number: CP052272), BL8800/BABL2880_pESBL (99.98%, a 75-kb region of *K. pneumoniae*69 p69-1, Genbank accession number: CP025457; data not shown).

pBL849_ESBL (89 kb) was highly similar to some sections of the p4_1_2.2 plasmid from *K. pneumoniae* strain 4/1-2 (99.98%, 96 kb, Genbank accession number: CP023841), isolated in Sweden in 2018 including evidence of rearrangements between regions. pBL12125_ESBL and pBL12456_ESBL (127 kb) were both comparable at 99.95% to part of pG747, a 151 kb plasmid isolated from the *K. pneumoniae* strain G747 in Maryland (USA) in 2018 (Genbank accession number: CP034137). pBA3783_ESBL (135 kb) was highly comparable (99.97%) to part of the p2K157 plasmid (157 kb) isolated from the *K. pneumoniae* strain KP69 (Genbank accession number: CP054291). pBL13802_ESBL (189 kb) was almost identical (99.99%) to part of a 180-kb plasmid that was present in a *K. pneumoniae* strain named *K. pneumoniae*_Goe_588–1 (Genbank accession number: CP018693), which was isolated in Germany in 2016. Also, pBL12125/456_ESBL, pBU19801_ESBL and pBL849_ESBL shared a common set of 7 ARGs including *aph(3″)-Ib* (or *strA*), *aph(6)-Id* (or *strB*), *bla*_TEM-1B_, *bla*_CTX-M-15_, *bla*_OXA-1_, and *aac(6′)-Ib-cr* (data not shown).

## Discussion

Understanding the evolution and spread of MDR bacteria is essential to countering the serious global threat posed by these organisms. The presence of ARGs on mobile genetic elements, strongly increases the risk of transferring resistance between different bacterial genera, including among those established in hospital environments. The development of novel sequencing methods and user-friendly web-based tools that fully characterize plasmids found in MDR pathogens is crucial for detecting and tracking the spread of AMR determinants across communities and preventing outbreaks.

Here, we characterized the plasmid structure in a collection of *bla*_NDM-1_ and *bla*_OXA-48_-harboring MDR *K. pneumoniae* isolated in Karachi, Pakistan using a hybrid NGS approach combining Nanopore long-read sequencing data with Illumina short-reads data. We also isolated *bla*_NDM-1_-plasmids of interest *via* conjugation to study correlation between plasmid-targeted AMR profiles and their associated susceptibility profiles. The genotypic AMR profiles of the *E. coli* transconjugants harboring only *bla*_NDM-1_-plasmids highlights the impact of *bla*_NDM-1_ and ARGs present on that same plasmid and co-transferred.

Several different types of plasmids associated with the *Enterobacteriaceae* have been reported to harbor *bla*_NDM_, including IncA/C, IncFII subtypes, IncH types, IncL/M, IncN and IncX (Ho et al., 2012; Partridge and Iredell, 2012; Carattoli, 2013). The variety of incompatibility groups and pMLST identified in our plasmids collection (IncC (types 1 and 3), IncFIA (type 26) IncFIB, IncFII (types K1, K2, K7, K9), IncHI1B, and IncL) are consistent with this observation.

All seven *bla*_NDM-1_-plasmids identified in our study were unique, however plasmids from isolates BL12125/BL12456 harbored the same IncC3 plasmid. As discussed previously (Lomonaco et al., 2018), BL12125 and BL12456 were identified as related strains ST14 sharing the same AMR profile, the same AST phenotype, and the same plasmid incompatibility group and plasmid type (IncC3). In both cases, *bla*_NDM-1_ is surrounded by a duplication of a set of ARGs (*bla*_OXA-10_, *aadA1*, and *sul1* including two copies of IS*CR1*) suggesting insertion events. A common IncL *bla*_OXA-48_-plasmid was also found to be shared by isolates BL8800/BA2880. Long read sequencing confirmed that the plasmids identified in BL12125 and BL12456 are identical. Additionally, BL8800 and BA2880 were found to carry the same *bla*_ESBL_-plasmid (IncFIIK9; 6 ARGs) and IncL *bla*_OXA-48_-plasmid, suggesting again that these 2 pairs of isolates are equivalent but obtained from different sites of infection in a single patient as previously reported by Lomonaco et al. (2018).

The *bla*_NDM-1-_plasmids isolated from BU19801 (ST307; IncFIIK2) and BA3783 (ST14, IncFIIK2) were 100% identical except for the insertion of a 746-bp truncated IS*Ec15* region on pBA3783_NDM, suggesting that the genetic rearrangements did not impact AMR profiles. However, both isolates BU19801 and BA3783 do share the same plasmid incompatibility group and type (IncFIIK2), AMR profiles, and AST phenotypes which strongly suggests that those two *K. pneumoniae* isolates may have previously shared the exact same plasmid.

Long-read technology has significantly enhanced the usefulness of NGS data to accurately predict AMR profiles, detect the presence of similar plasmids in different isolates, and identify genetic rearrangement events. For instance, pBL849 [IncFIB(pNDM-Mar)/IncHI1B(pNDM-MAR), pBL12125/456 (IncC3)] shared 3 AMR modules that lead to the same AMR profiles but carried both different non-AMR regions and incompatibility groups. This observation suggests that their respective plasmids are different.

pMS84_NDM and pBL13802_NDM were similar to two *bla_NDM-1_*-plasmids described previously. pMS84_NDM, which was IncC1-positive, shared 100% identity with an IncA/C2 *bla_NDM-1_*-plasmid (GenBank accession number: C050164) isolated from a *K. pneumoniae* strain from Hong-Kong in 2020. The IS*Ec23* element found in pMS84_NDM suggests that their respective plasmids are related. pBL13802_NDM, which was IncFII(pKPX1)/type 1-positive, shared 99.98% identity with the pKPX-1 plasmid identified from a *K. pneumoniae* clinical isolate obtained from a rectal swab in Taiwan in 2016 (Huang et al., 2013). BL13802_pNDM and pKPX-1, shared a ~130-kb region comprising a stretch of AMR determinants, but a 121-kb region neighboring *bla*_NDM-1_, and filled with a succession of IS elements, was missing from pBL13802_NDM. The KPX strain originated from a Taiwanese patient with a hospitalization history in New Delhi (Huang et al., 2013), which may suggest that those two plasmids are also related.

The *bla*_OXA-48_-plasmids (IncL) identified in this study were identical to the pRJ119-2 plasmid reported in 2020 as the first *bla*_OXA-48-_plasmid in China (Genbank accession number: KX636096). Finally, while some large AMR regions have been reported, all *bla*_ESBL_-plasmids identified in this study have not been previously described. The AMR features were observed in multiple rearrangements which underscores the complexity of molecular background and importance of mobility of ARGs *via* horizontal transfer.

All *bla*_NDM-1_-positive *E. coli* transconjugants were resistant to most β-lactams, and all aminoglycosides while these transconjugants were susceptible to CIP, SXT, TET, and CHL. All *E. coli* transconjugants were susceptible to ATM as expected for NDM producers except for Ec_BL12125/456 due to the presence of *bla*_OXA-10_ on the *bla*_NDM-1_-plasmid. All *bla*_OXA-48_-positive *E. coli* transconjugants were resistant to ampicillin, β-lactams/β-lactamase inhibitor combinations (AMC, TZP, and SAM), but susceptible to fluoroquinolones, other β-lactams and aminoglycosides.

Overall, the NGS/RF-phenotypes-based approach performed well in comparison with BMD for *E. coli* transconjugants except for a few instances: CRO, ATM, and CHL. The potential hydrolysis of CRO (MIC >32 μg/ml) by the NDM enzyme was apparently not accounted for by ResFinder. The OXA-48 enzyme, which is not a particularly efficient carbapenemase often referred to “phantom menace” or “hidden threat” (Poirel et al., 2012; Bakthavatchalam et al., 2016) is difficult to detect and often missed by routine diagnostics was detected by ResFinder both genotypically and phenotypically. Both *K. pneumoniae* isolates harboring the *cmlA5* gene (BL12125/456) were classified as susceptible by ResFinder and BMD. In both study isolates, two important components of the class 1 integron described by Revathi et al. (2013) were altered: (1) truncated integrase (*intI1*) and (2) absence of the Pc promoter for the *cmlA5* gene cassette. This finding suggests that *cmlA5* may not be expressed in this integron. Lastly, all *E. coli* transconjugants carrying *qnrB/S* or *aac(6′)-Ib-cr* were susceptible to fluoroquinolones (CIP), which implies that the high-level fluoroquinolone resistance phenotypes observed in our previous study (Lomonaco et al., 2018) are most likely due to chromosomal mutations on genes encoding DNA gyrase and topoisomerases IV, respectively.

It has been well-established that genetic-based screening approaches informed by high-quality sequencing data have potential to be useful to rapidly detect the most common and well-described mechanisms of resistance which correlate with phenotypes for the major classes of antimicrobials. However, the current bioinformatics landscape lacks a standardized and well-curated database with expert-defined rules for AMR gene inclusion/exclusion (e.g., truncated genes). Moreover, our findings reiterate that the presence/detection of ARGs does not necessarily yield a resistant phenotype, as observed for *qnrB1, aac(6′)-Ib-cr* and *cmlA5*.

In this study, bacterial conjugation provided additional information on phenotypes of targeted plasmids but one of the limitations is that plasmids over ~200 kb may be difficult to transfer (e.g., BL249, 277 kb). This study did not aim to investigate the transfer of plasmids between isolates, since this collection did not include all carbapenemase-positive *Enterobacteriaceae* samples from the same period. However, this study highlights how comprehensive characterization of AMR plasmids may offer insights to rapidly respond to outbreaks and help early implementation of effective infection controls procedures.

The use of ONT/Illumina sequencing is crucial for accurate and complete plasmid characterization in research laboratories, but ONT sequencing could also provide preliminary results within healthcare institutions to inform and fortify infection control measures while waiting for AST results that would confirm expression of the ARGs detected by NGS. Nanopore offers a specific workflow for plasmids that has the potential to identify pathogens, AMR determinants, and plasmid classification and/or structural features within minutes (Tamma et al., 2019; Neal-McKinney et al., 2021).

Further comprehensive plasmid analysis including a hybrid approach can be performed for epidemiologic purposes. Real-time sequencing approaches could enable MDR plasmid characterization and analysis as new isolates are collected. To maximize impact, the data could be used to build a curated AMR-based plasmid database, that combines AMR profiles and metadata. Overall, such a resource would provide scientists and clinicians a powerful infection control surveillance system that tracks plasmid-based AMR transmission in real time.

This study highlights the complexity and diversity of molecular background of pathogens that produce carbapenemases along with ESBL or AmpC ß-lactamases, which are challenging for clinicians. The spread of plasmid-mediated AMR determinants between pathogens emerging as MDR bacteria is a major threat worldwide, and rapid tracking of such pathogens is warranted. This study emphasizes the value of rapid, real-time sequencing of AMR plasmids to predict NGS-based AST profiles, and to provide insights on their dissemination especially during outbreaks.

## Data availability statement

The datasets presented in this study can be found in online repositories. The names of the repository/repositories and accession number(s) can be found in the article/Supplementary material.

## Author contributions

CL, MC, MH, EK, KA, DH, SP, and DS contributed to conception and design of the study. CL, BC, DF, and MC performed all lab experiments. AC and LR performed the bioinformatics data analysis. CL wrote the first draft of the manuscript. All authors contributed to the article and approved the submitted version.

## Funding

This project was supported by the Department of Homeland Security through an interagency agreement HSHQPM-14-X-00116 with the Centers for Disease Control and Prevention. MC and MH received additional support from the U.S. National Institutes of Health grants R21 AI139947 and R01 AI150941. The findings and conclusions in this report are those of the authors and do not necessarily represent the views or official positions of the Department of Health and Human Services, Centers for Disease Control and Prevention, U.S. Food and Drug Administration and the Department of Homeland Security. The use of trade names is for identification only and does not imply endorsement.

## Conflict of interest

The authors declare that the research was conducted in the absence of any commercial or financial relationships that could be construed as a potential conflict of interest.

## Publisher’s note

All claims expressed in this article are solely those of the authors and do not necessarily represent those of their affiliated organizations, or those of the publisher, the editors and the reviewers. Any product that may be evaluated in this article, or claim that may be made by its manufacturer, is not guaranteed or endorsed by the publisher.

## Footnotes

^1^https://cge.cbs.dtu.dk/services/ResFinder/

^2^https://cge.food.dtu.dk/services/PlasmidFinder/

^3^https://cge.food.dtu.dk/services/pMLST/
