# Supplementary material for: Investigation of multidrug-resistant plasmids from carbapenemase-producing Klebsiella pneumoniae clinical isolates from Pakistan
Source: Front Microbiol. 2023 Jun 29;14:1192097. doi: 10.3389/fmicb.2023.1192097 (PMC10340517; doi:10.3389/fmicb.2023.1192097)
Supplement: Supplementary file 1 [file Data_Sheet_1.docx]

*Antimicrobial agents not tested by BMD.

**Figure S1: Distribution of plasmid-mediated AMR determinants identified in MDR *Klebsiella pneumoniae* clinical isolates.**

| **ID Name** | ***bla*_NDM-1_** | ***bla*_OXA-48_** | ***bla*_TEM-1B_** | ***bla*_CTX-M-15_** | ***bla*_OXA-1, 10_** | ***bla*_CMY-6, -16_** | **AMP, AMC, SAM, TZP** | **FOX** | **CTX** | **CAZ** | **CRO** | **FEP** | **ATM** | **IPM** | **DOR** | **MEM** | **Phenotype** |
| --- | --- | --- | --- | --- | --- | --- | --- | --- | --- | --- | --- | --- | --- | --- | --- | --- | --- |
| **pBL849** |  |  | x | x | 1 ; 10 |  | R | R | R | R | R | R | R | R | R | R | RF-based |
| **pBU19801** |  |  | x | x | 1 |  | R | R | R | R | R | R | R | R | R | R | RF-based |
| ***Ec_pBU19801_NDM*** |  |  |  |  |  |  | >32 | >16 | >64 | >128 | >32 | >32 | ≤2 | 4 | 4 | 8 | AST |
|  |  |  |  |  |  |  | R | R | R | R | NR | R | NR | R | R | R | RF-based |
| **pMS84** |  |  |  |  |  | 6 | R | R | R | R | NR | R | NR | R | R | R | RF-based |
| ***Ec_pMS84_NDM*** |  |  |  |  |  | 6 | >32 | >16 | >64 | >128 | >32 | >32 | ≤2 | 4 | 4 | 8 | AST |
|  |  |  |  |  |  |  | R | R | R | R | NR | R | NR | R | R | R | RF-based |
| **pBL12125/pBL12456** |  |  | x | x | 1 ; 10 | 16 | R | R | R | R | R | R | R | R | R | R | RF-based |
| ***Ec_pBL12125/BL12456_NDM*** |  |  |  |  | 10 | 16 | >32 | >16 | >64 | >128 | >32 | >32 | 32 | 4 | 4 | 4 | AST |
|  |  |  |  |  |  |  | R | R | R | R | NR | NR | R | NR | NR | NR | RF-based |
|  |  |  |  |  |  |  | R | R | R | R | NR | R | R | R | R | R | RF-based |
| **pBA3783** |  |  |  | x | 1 |  | R | R | R | R | R | R | R | R | R | R | RF-based |
| ***Ec_pBA3783_NDM*** |  |  |  |  |  |  | >32 | >16 | >64 | >128 | >32 | 16 | ≤2 | 2 | 4 | 4 | AST |
|  |  |  |  |  |  |  | R | R | R | R | NR | R | NR | R | R | R | RF-based |
| **pBL13802** |  |  |  | x |  |  | R | R | R | R | R | R | R | R | R | R | RF-based |
| ***Ec_pBL13802_NDM*** |  |  |  |  |  |  | >32 | >16 | >64 | >128 | >32 | >32 | ≤2 | 4 | >8 | 4 | AST |
|  |  |  |  |  |  |  | R | R | R | R | NR | R | NR | R | R | R | RF-based |
| **pBA2664** |  |  |  |  |  |  | R | NR | NR | NR | NR | NR | NR | R | R | NR | RF-based |
| ***Ec_pBA2664_OXA-48*** |  |  |  |  |  |  | >32 | 8 | 1 | 1 | ≤1 | ≤0.5 | ≤2 | 2 | 2 | 1 | AST |
|  |  |  |  |  |  |  | R | NR | NR | NR | NR | NR | NR | R | R | NR | RF-based |
| **pBL8800/pBA2880** |  |  | x |  | 1 |  | R | NR | NR | NR | NR | R | NR | R | R | NR | RF-based |
| ***Ec_pBL8800_OXA-48*** |  |  |  |  |  |  | >32 | 8 | 1 | 1 | ≤1 | ≤0.5 | ≤2 | 1 | 2 | 1 | AST |
|  |  |  |  |  |  |  | R | NR | NR | NR | NR | NR | NR | R | R | NR | RF-based |

**Table S3: Illumina/Nanopore-based antimicrobial resistance determinant profiles and AST results for β-lactams.**

Ampicillin (AMP), ampicillin+sulbactam (SAM), amoxicillin+clavulanate (AMC), piperacillin/tazobactam (TZP), cefazolin (CZ), cefoxitin (FOX), cefotaxime (CTX), ceftazidime (CAZ), ceftriaxone (CRO), cefepime (FEP), aztreonam (ATM), imipenem (IPM), doripenem (DOR), meropenem (MER).

An “x” indicates the presence of a gene as determined by ResFinder 4.0 (RF).

The presence of *bla*_NDM-1_, and *bla*_OXA-48_ plasmids is highlighted in gray. The AMR genes present on *bla*_ESBL,_ and *bla*_NDM-1_-plasmids are highlighted dark blue and pink, respectively. *bla*_OXA-1_ and *bla*_OXA-10_ are identified on *bla*_ESBL_ and *bla*_NDM-1_-plasmids, respectively.

R: Resistant; NR: No Resistance.

|  |  | |  | **Plasmid-mediated Aminoglycosides AMR Genes** | | | | | | | | | | | **Phenotype (MIC, ug/mL)** | | |
| --- | --- | --- | --- | --- | --- | --- | --- | --- | --- | --- | --- | --- | --- | --- | --- | --- | --- |
| **ID Name** | ***bla*_NDM-1_** | ***bla*_OXA-48_** | | ***aac(3)-IIa/d*** | ***aac(6')-Ib*** | ***aac(6')-Ib-cr*** | ***armA*** | ***rmtC*** | ***rmtF*** | ***aadA1/A2*** | ***aph(3')-Ia*** | ***aph(3')-VI*** | ***aph(3'')-Ib=strA*** | ***aph(6)-Id=strB*** | **AMK** | **GEN** | **TOB** |
| ***pBL849*** |  |  | | a |  | x | x |  |  |  |  |  | x | x | R (>64) | R (>16) | R (>16) |
| ***pBU19801*** |  |  | | a |  | x | x |  |  |  |  |  | x | x | R (>64) | R (>16) | R (>16) |
| ***Ec_pBU19801_NDM*** |  |  | |  |  | x | x |  |  |  |  |  | x | x | R (>64) | R (>16) | R (>16) |
| ***pMS84*** |  |  | |  |  | x |  | C |  |  |  |  |  |  | R (>64) | R (>16) | R (>16) |
| ***Ec_pMS84_NDM*** |  |  | |  |  | x |  | C |  |  |  |  |  |  | R (>64) | R (>16) | R (>16) |
| ***pBL12125/456*** |  |  | | a |  | x | x |  |  | A1 |  |  | x | x | R (>64) | R (>16) | R (>16) |
| ***Ec_pBL12125/456_NDM*** |  |  | |  |  |  | x |  |  | A1 |  |  |  |  | R (>64) | R (>16) | R (>16) |
| ***pBA3783*** |  |  | |  |  | x | x |  |  |  |  |  | x | x | R (>64) | R (>16) | R (>16) |
| ***Ec_pBA3783_NDM*** |  |  | |  |  | x | x |  |  |  |  |  | x | x | R (>64) | R (>16) | R (>16) |
| ***pBL13802*** |  |  | | a |  | x |  |  | F | A2 | x |  |  |  | R (>64) | R (>16) | R (>16) |
| ***Ec_pBL13802_NDM*** |  |  | |  |  | x |  |  | F |  |  |  |  |  | R (>64) | R (>16) | R (>16) |
| ***pBA2664*** |  |  | | d |  |  |  |  |  |  |  |  |  |  | R (8) | R (8) | R (8) |
| ***Ec_pBA2664_OXA-48*** |  |  | |  |  |  |  |  |  |  |  |  |  |  | NR (4) | NR (1) | NR (< 0.5) |
| ***pBL8800/BABL2880*** |  |  | | a |  | x |  |  | F |  |  |  | x |  | R (>64) | R (>16) | R (>16) |
| ***Ec_pBL8800/BABL2880_OXA-48*** |  |  | |  |  |  |  |  |  |  |  |  |  |  | NR (2) | NR (1) | NR (< 0.5) |

**Table S4: Illumina/Nanopore-based antimicrobial resistance determinant profiles and AST results for aminoglycosides.**

Amikacin (AMK), gentamicin (GEN), tobramycin (TOB).

An “x” indicates the presence of a gene as determined by ResFinder 4.0 (RF). The presence of *bla*_NDM-1_, and *bla*_OXA-48_ plasmids is highlighted in gray.

The AMR genes present on *bla*_ESBL_ and *bla*_NDM-1_-plasmids with 100% identity are highlighted in dark blue and pink, respectively. The AMR genes identified with 95-99.9% are highlighted in light pink, blue or green.

R: Resistant; NR: No Resistance.

|  |  |  | **AMR genes (RF)** | | | **Phenotypes (RF, MIC – ug/mL)** |
| --- | --- | --- | --- | --- | --- | --- |
| **Isolates ID** | ***bla*_NDM-1_** | ***bla*_OXA-48_** | ***aac(6’)-Ib-cr**** | ***aac(6’)-Ib-cr*** | ***qnrB/S*** | **CIP** |
| ***pBL849*** |  |  | - | x | S1 | R (>32) |
| ***pBU19801*** |  |  | x | x | - | R (>32) |
| ***Ec_pBU19801_Tc1*** |  |  | x | - | - | R (< 0.25) |
| ***pMS84*** |  |  | x | - | - | R (>32) |
| ***Ec_pMS84_Tc1*** |  |  | x | - | - | R (< 0.25) |
| ***pBL12125/456*** |  |  | - | x | B1 | R (3) |
| ***Ec_pBL12125/456_Tc1*** |  |  | - | - | - | NR (< 0.25) |
| ***pBA3783*** |  |  | x | x | B1 | R (8) |
| ***Ec_pBA3783_Tc1Tic*** |  |  | x | - | - | R (0.25) |
| ***pBL13802*** |  |  | x | - | - | R (>32) |
| ***Ec_pBL13802_Tc1Tic*** |  |  | x | - | - | R (< 0.25) |
| ***pBA2664*** |  |  | x | - | - | R (>32) |
| ***Ec_pBA2664_Tc1Tic*** |  |  | - | - | - | NR (< 0.25) |
| ***pBL8800/pBABL2880*** |  |  | x | x | - | R (>32) |
| ***Ec_pBL8800/BABL2880_Tc1Tic*** |  |  | - | - | - | NR (< 0.25) |

**Table S5: Illumina/Nanopore-based antimicrobial resistance determinant profiles and AST results for fluoroquinolones.**

CIP : ciprofloxacin.

AMR genes present on *bla*_NDM-1_, and *bla*_ESBL_-plasmids are highlighted in pink and blue, respectively.

An “x” indicates the presence of a gene as determined by ResFinder 4.0 (RF).

Susceptible, Intermediate and Resistant results by BMD are highlighted in green, yellow, and red, respectively.

*aac(6’)-Ib-cr* = DQ303918 (600 bp) – 100%; *aac(6’)-Ib-cr** = EF636461 (519 bp) – 99.61%

R: Resistant; NR: No Resistance.

|  |  |  | **SXT** | | | | **TET** | | **CHL** | | |
| --- | --- | --- | --- | --- | --- | --- | --- | --- | --- | --- | --- |
| **ID name** | ***bla*_NDM-1_** | ***bla*_OXA-48_** | *dfrA1, A12, A14* | *sul1, -2* | | **RF-based (MIC, ug/mL)** | *tetA* | **RF-based (MIC, ug/mL)** | *cmlA* | *catA1* | **RF-based (MIC, ug/mL)** |
| ***pBL849*** |  |  | A1 | 1* | 1 | NT (>8) | x | R | A5* | A1 | R (>16) |
| ***pBU19801*** |  |  | A14 | 1 | 2 | NT (>8) | - | NR | - | - | R (8) |
| ***Ec_pBU19801_NDM*** |  |  |  | 1 | - | NT (<0.5) | - | NR (<2) | - | - | NR (4) |
| ***pMS84*** |  |  |  | 1 | - | NT (2) | - | NR | - | - | NR (8) |
| ***Ec_pMS84_NDM*** |  |  |  | 1 | - | NT (<0.5) | - | NR (<2) | - | - | NR (4) |
| ***pBL12125/456*** |  |  | A14 | 1 | 2 | NT (>8) | x | R | A5* |  | R (8) |
| ***Ec_pBL12125/456_NDM*** |  |  |  | 1 |  | NT (<0.5) | - | NR (<2) | A5* | - | R (8) |
| ***pBA3783*** |  |  | A14 | 1 | - | NT (>8) | - | NR | A5* |  | R (8) |
| ***Ec_pBA3783_NDM*** |  |  |  | 1 | - | NT (<0.5) | - | NR (<2) | - | - | NR (4) |
| ***pBL13802*** |  |  | A12 | - | 1 | NT (>8) | - | NR | - | - | NR (>16) |
| ***Ec_pBL13802_NDM*** |  |  | - | - | - | NT (1) | - | NR (<2) | - | - | NR (4) |
| ***pBA2664*** |  |  | - | - | 1 | NT (<0.5) | - | NR | - | - | NR (16) |
| ***Ec_pBA2664_OXA-48*** |  |  | - | - |  | NT (<0.5) | - | NR (<2) | - | - | NR (4) |
| ***pBL8800/BABL2880*** |  |  | - | - | 2 | NT (<0.5) | - | NR | - |  | R (8) |
| ***Ec_pBL8800/BABL2880_OXA-48*** |  |  | - | - | - | NT (< 0.5) | - | NR (<2) | - | - | NR (4) |

**Table S6: Illumina/Nanopore-based antimicrobial resistance determinant profiles and AST results for cotrimoxazole, tetracycline, and chloramphenicol.**

SXT : cotrimoxazole, TET: tetracycline, CHL : chloramphenicol.

AMR genes present on *bla*_NDM-1_, and *bla*_ESBL_-plasmids are highlighted in blue, and pink, respectively.

Susceptible, Intermediate and Resistant results by BMD are highlighted in green, yellow, and red, respectively.

*cmlA1* was identified with 99.6% similarity by RF but was identified *cmlA5* using BLAST.

R: Resistant; NR: No Resistance.
